# Effect of crosslinking concentration on properties of 3-(trimethoxysilyl) propyl methacrylate/*N*-vinyl pyrrolidone gels

**DOI:** 10.1186/s13065-018-0379-4

**Published:** 2018-02-13

**Authors:** Ameen Hadi Mohammed, Mansor B. Ahmad, Nor Azowa Ibrahim, Norhazlin Zainuddin

**Affiliations:** 1 Department of Chemistry, Faculty of Science, Universiti Putra Malaysia, 43400 Serdang, Selangor Malaysia; 2 Department of Chemistry, College of Science for Women, University of Baghdad, Al Jadria, Baghdad, 10071 Iraq

**Keywords:** Hydrogels, Poly 3-(trimethoxysilyl) propyl methacrylate/*N*-vinyl pyrrolidone, Crosslinking density, Thermal properties, Oxygen permeability

## Abstract

**Background:**

The incorporation of two different monomers, having different properties, in the same polymer molecule leads to the formation of new materials with great scientific and commercial importance. The basic requirements for polymeric materials in some areas of biomedical applications are that they are hydrophilic, having good mechanical and thermal properties, soft, and oxygen-permeable.

**Results:**

A series of 3-(trimethoxysilyl) propyl methacrylate/*N*-vinyl pyrrolidone (TMSPM/NVP) xerogels containing different concentration of ethylene glycol dimethacrylate (EGDMA) as crosslinking agent were prepared by bulk polymerization to high conversion using BPO as initiator. The copolymers were characterized by FTIR. The corresponding hydrogels were obtained by swelling the xerogels in deionized water to equilibrium. Addition of EGDMA increases the transparency of xerogels and hydrogels. The minimum amount of EGDMA required to produce a transparent xerogel is 1%. All the Swelling parameters, including water content (EWC), volume fraction of polymer (*ϕ*_2_) and weight loss during swelling decrease with increasing EGDMA. Young’s and shear modulus (E and G) increase as EGDMA increases. The hydrogels were characterized in terms of modulus cross-linking density (*v*_*e*_ and *v*_*t*_) and polymer-solvent interaction parameters (χ). Thermal properties include TGA and glass transition temperature (T_g_) enhance by adding EGDMA whereas the oxygen permeability (P) of hydrogels decreases as water content decrease.

**Conclusions:**

This study prepared and studied the properties for new copolymer (TMSPM-co-NVP) contains different amounts of (EGDMA). These copolymers possess new properties with potential use in different biomedical applications. The properties of the prepared hydrogels are fit with the standard properties of materials which should be used for contact lenses.

## Introduction

Hydrogels are hydrophilic polymeric networks that absorb water from 10% to hundreds times their dry weight and are insoluble in water because of the presence of a three-dimensional network [1]. Existing hydrogel materials, which are claimed to be useful in some important biomedical applications, are derived almost exclusively from hydrophilic monomers such as 2-hydroxyethyl methacrylate, glycerol methacrylate or *N*-vinyl pyrrolidone. These hydrogels, in general, have water contents ranging from 38 to 75%. The water content and modulus of hydrogels depend on the nature of monomers and crosslinking density [2, 3].

Although polymeric hydrogels have good biocompatibility, their mechanical strength on swelling is very poor. For getting materials combining biocompatibility with a good mechanical strength, two methods are used: Copolymerization of hydrophobic monomers with hydrophilic monomers or using cross-linking agent [4].

Because of the high biocompatibility and low toxicity, the utilization of hydrogels as biomaterials has recently gained great importance. Today the major fields of hydrogels applications involve: injectable polymers, ophthalmic applications, topical applications as wound and burn dressings, dental applications, drug delivery systems [5], blood compatible materials [6], implants [7, 8], and stimuli responsive systems.

Accordingly, it would be highly useful in a number of medical applications to provide a polymeric material having increased hydrophilicity, softness after hydration, mechanical strength and oxygen permeability. Siloxane derivative compounds have been used in hydrogels for biomedical applications [9]. Copolymers of these compounds with different monomers present interesting permeation properties and have been proposed as potential polymers for biomedical applications. More specifically, it has been suggested that a higher permeability of oxygen can be achieved, due to the considerable contribution of the (–Si–O–) bonds and solubility of oxygen in the film [10].

Poly vinylpyrrolidone (PNVP) is of prime importance among other macromolecular compounds. A combination of practically valuable characteristics of PNVP: solubility in water and in most organic solvents, absence of toxicity and biological compatibility. Therefore, copolymers of *N*-vinylpyrrolidone (NVP) have a still wider set of valuable characteristics. The copolymerization reactions allow modification of PNVP characteristics and therefore, acquire growing practical importance [11, 12].

It is clear that the combination of a hydrophilic group with a siloxane group in the polymer may give a suitable biomedical material, and it may have the following properties: chemically stable compound, transparent, a moderate elastic modulus, soft when hydrated with a good degree of swelling and oxygen permeable. However a copolymer of these two compounds shows a range of incompatibility. This is interphase with a third component (ethylene glycol dimethacrylate EGDMA) in addition to its basic function as a cross-linking agent.

The aim of this work is the preparation of gels by copolymerization a hydrophilic monomer (NVP) with a hydrophobic monomer (TMSPM). This work also studies the influence of crosslinking content (EGDMA) on the properties of xerogels and hydrogels such as, optical homogeneity, swelling behavior, mechanical properties, thermal properties and oxygen permeability.

## Experimental section

### Materials

Commercial samples of monomers (NVP) and 3-(trimethoxysilyl) propyl methacrylate (TMSPM) from Aldrich chemical were purified by passing them through an aluminum oxide (Al_2_O_3_) column (2.5 × 15 cm) until colorless products were obtained. The initiator (BPO) was recrystallized from chloroform to dried in a vacuum. (EGDMA), dichlorodimethylsilane, methanol and deionized water were used as received.

### Preparation of xerogels

Sample ampoules have enough surface area and 13 mm diameter, were used to be suitable for the isothermal condition during the polymerization. The ampoules were siliconized with a 2% solution of dichlorodimethylsilane in chloroform and kept in an air oven for 1 day at 75 °C to facilities the removing of polymer rods. Monomers mixture (3 g TMSPM/7 g NVP) containing 0.5% (BPO) as initiator and different amount of EGDMA as crosslinker (0, 0.5, 1, 1.5 and 2%) was made up in a small stopper flask. The mixture was stirred for 15 min, and then transferred to the glass ampoules which have been siliconized previously. The contents of tubes were purged with nitrogen for (15 min) prior to the reaction in order to remove all oxygen. The glass ampoules were placed in a water bath at 50 °C, and allowed to polymerization for a specified time (2 days). The temperature is then raised and the tubes are placed in an oven 70 °C for another 1 day. At the end of this time, polymerization is normally completed, after which the polymerized rods were removed from the tubes. The rods were then post cured for 1 day at 70 °C to complete the polymerization process and relieve any mechanical stresses present. The resulting xerogels were cut into a disk and put in methanol to remove the residual unreacted monomers. All discs were dried exhaustively in an oven at 35 °C to constant weight. Then the efficiency of synthesis was determined by using gel fraction equation:

1 $$G = \frac{{W_{p} }}{{W_{m} }} \times 100$$

where W_p_ is the weight of the dried disc and W_m_ is the weight of the two monomers mixture, for all the compositions the gel fractions were > 98%.

**Scheme 1:**
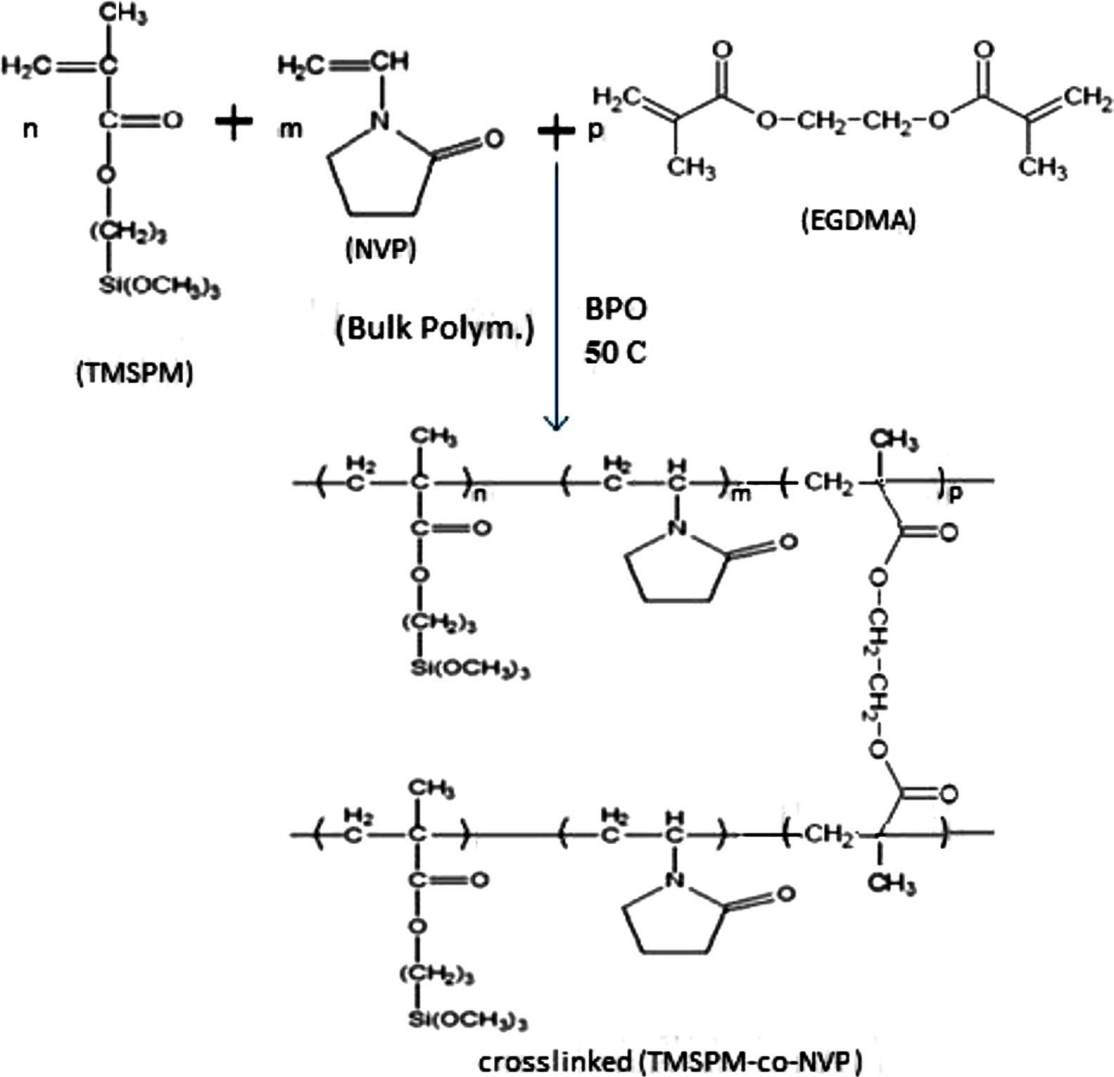
Schematic illustration of the process formation of crosslinked (NVP-*co*-TMSPM) from NVP to TMSPM as monomers and EGDMA as crosslinker

The reaction is shown in the following Scheme  1.

### Swelling studies

The swelling of the discs was carried out at room temperature 25 °C. The known weight and diameters of dried discs were put in sample vials (50 ml). The swelling time was counted from when the deionized water was added into the vial. At regular time intervals, the swollen discs were taken out using tweezers, and the excess water on the surface of the discs was removed by wiping with the edge of Whitman No. 1 qualitative filter paper. They were weighted and returned to the vials immediately. The water content (EWC), reduction in the weight of the xerogels, extension ratio (ER) and volume fraction of polymer ($\phi_{2}$) were calculated as [13]:

2 $$EWC \% = \frac{{\left( {W_{s} - W_{d} } \right)}}{{W_{s} }} \times 100$$

3 $$\text{Weight loss during swelling} = \frac{{\left( {W_{0} - W_{d} } \right)}}{{W_{0} }} \times 100$$

where W_0_, W_s_ and W_d_ are the weights of the xerogel, swollen sample after 30 days fully hydrated, and after drying in an oven at 40 °C for (48 h.), respectively.

4 $$ER = \frac{d}{{d_{0} }}$$

5 $$\phi_{2} = \left( {\frac{{d_{0} }}{d}} \right)^{3}$$

where, d_0_ and d are the diameters of dry and fully hydrated discs, respectively. Thus, the volume fraction of water ($\phi_{1}$) in the hydrogel at equilibrium is equal to ($1 - \phi_{2}$).

### Compression measurements

An Instron 3366 machine analyzer was used for compression strain–stress testing. The crosshead speed was set at a fixed rate of 2 mm/min, and the load was run until the sample was broken. For testing, the hydrogels were cut into strips with dimensions of $\sim$ (25 mm in length, 5 mm in width and 2 mm in thickness).

Young’s modulus E for the hydrogels was obtained as the slopes in plots of the stress (τ) versus strain (λ − 1), as follows:

6 $$\tau = E\left( {\lambda - 1} \right)$$

where τ is the applied force per unit area of hydrogel and λ is the ratio of deformed length (l) of hydrogel to its undeformed (l_0_). The effective crosslinking density (ν_e_) of hydrogels can be obtained from the compression-strain measurements via the kinetic theory of rubbery elasticity [14].

7 $$\tau = G \left( {\lambda - \lambda^{ - 2} } \right)$$

8 $$G = RT\nu_{e} \phi_{2}^{1/3}$$

G can be obtained from the slope of the stress, τ, versus $\left( {\lambda - \lambda^{ - 2} } \right)$. In Eq. (7), *ϕ*_2_ is the volume polymer fraction, R is the gas constant (8.314 J/K/mol) and T is the absolute temperature. The polymer/solvent interaction parameter, χ, which represents the specific interaction between water and polymers, can be calculated from the Flory–Rehner equation [15].

9 $$ln\left( {1 - \phi_{2} } \right) + \phi_{2} + \chi \phi_{2}^{2} + \nu_{e} V_{1} \left( {\phi_{2}^{1/3} - 2 \phi_{2} f^{ - 1} } \right) = 0$$

In which V_1_ is the molar volume of water (18.05 × 10^−3^ dm^3^/mol at 298 °K) [16] and f is the functionality of the cross-linker agent. The molecular mass between cross-links, M_c_ can be calculated via Eq. (10), in which ρ is the density of the xerogl.

10 $$M_{c} = \rho /\nu_{e}$$

The theoretical cross-linking density $v_{t}$ was calculated from the following relationship:

11 $$v_{t} = Cf/2$$

where, C is the concentration of cross-linking agent with functionality *f*. Because $f$ = 4 for EGDMA [16], Eq. (11) is reduced to:

12 $$v_{t} = 2C$$

The values of C were calculated from the weight concentration of EGDMA by using (198.22 g/mol) as the molar mass of EGDMA and by taking the densities of the xerogels.

### Oxygen permeation evaluation

Stainless steel filter holder (Merck, Frankfurter, Darmstadt, Germany) was used for oxygen permeation experiments. Glass soap bubble flow meter was employed for measuring rate of permeate stream. Glass soap bubble flow meter is useful for measuring any gas flow rate and it gives accurate measurement [17, 18]. The gases below the surface of a soap bubble solution and the bubble moves up the flow meter. We time the leading edge of the bubble from one line to another. To ensure accuracy in our experiments, the gas permeation test was repeated two times in the steady state. Permeability across polymer matrix can be described as follows [19]:

13 $$(P/L) = Q/\left( {A \times \Delta P} \right)$$

where P is permeability, L is hydrogel thickness, Q is gas flow (at standard pressure and temperature), A is the hydrogel effective area, and ΔP is the differential partial pressure through the sample. The common unit of permeance is GPU and 1 GPU is equal to $1 \times 10^{4}$ barrier.

### Thermal properties

Thermal degradability of the polymer was studied by TGA using Perkin Elmer in a nitrogen atmosphere at a heating rate of 10 °C/min from 0 to 800 °C and glass transition temperature (T_g_) was determined using a DSC-Mettler calorimetric system.

## Results and discussion

### Characterization of copolymer

**Fig. 1:**
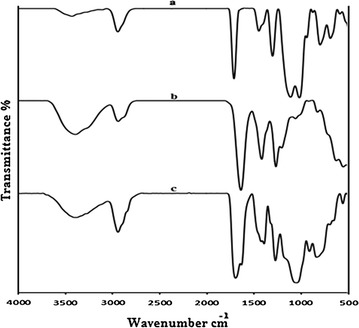
FTIR of: * a* PTMSPM, * b* PNVP, * c* TMSPM-co-NVP

The structure of TMSPM/NVP copolymer is confirmed by FITR as shown in Fig. 1. The absorption bands which appear in the FTIR spectra of the copolymer (Fig. 1c) belong to the stretching vibration in different functional groups of corresponding homopolymers (Fig. 1a, b). The absorption bands of TMSPM/NVP copolymer as follows: 2925 (alkane C–H), the carbonyl absorption of TMSPM observed at 1710 (ester C=O), 1650 (tertiary amide C=O), 1270 (amide C–N), 1075 (Si–O), 850/cm (Si–C) and (alkane C–H bending vibration) at about 1400/cm.

**Fig. 2:**
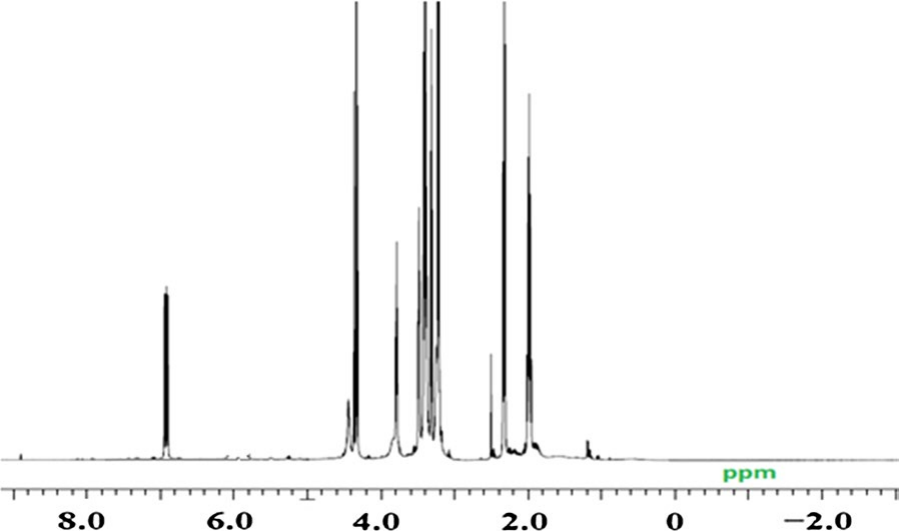
^1^H-NMR of TMSPM/VP copolymer

The ^1^H-NMR of the copolymers was recorded with a JOEL JMTC-500/54/SS (500 MHz) spectrometer using dimethylsulfoxide (DMSO) as the solvent and tetramethylsilane (TMS) as the internal standard. Figure 2 shows ^1^H-NMR spectrum of the copolymer. Methylene protons in NVP ring resonate in 2.5, 3.2 and 4.4 ppm, while CH_2_ protons for main chain backbone of monomers resonate at 1.8–2.4 ppm. CH proton main chain backbone of NVP resonates at 4.6 ppm. The signal corresponding to the protons of the methoxy groups linked to the silicon atom in TMSPM at about 3.5 ppm can be clearly observed. The ester and methyl groups in TMSPM resonate at 3.8 and 1.2 ppm, respectively. The stronger peak appearing at about 6.9 ppm could be attributed to the proton of =C–H.

### Optical homogeneity

**Fig. 3:**
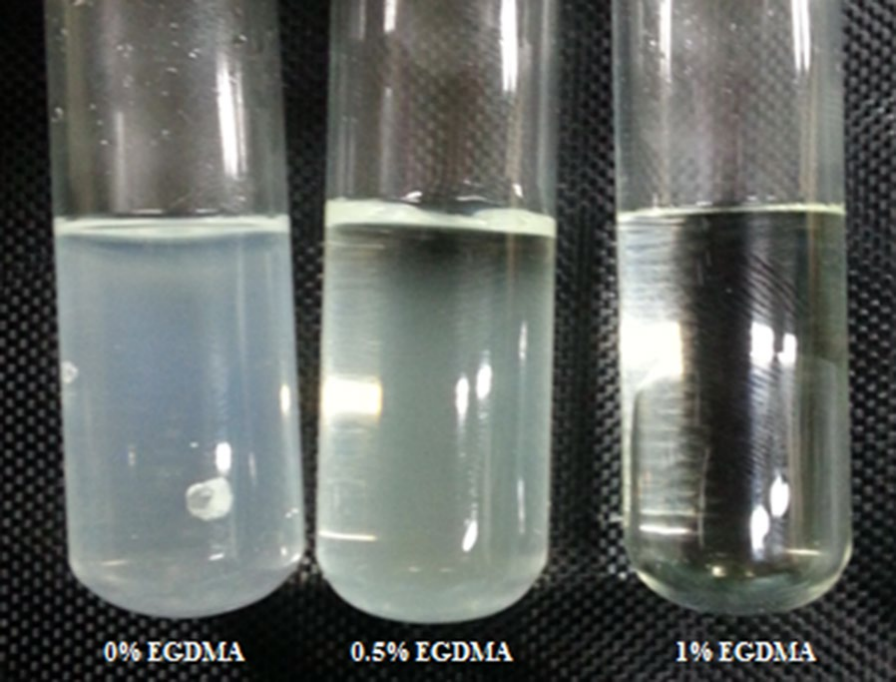
Photograph of some prepared xerogels with different optical clarity

**Fig. 4:**
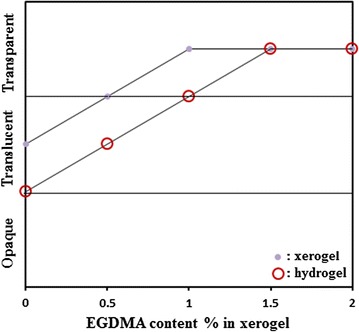
Optical homogeneity of TMSPM30/NVP70 xerogels and hydrogels with various amount of EGDMA

When the preparation process of xerogels and hydrogels was completed, a visual assessment of homogeneity and optical clarity was made. Figure 3 shows photograph of some prepared xerogels with different degree of optical clarity. The xerogel and hydrogel without EGDMA are translucent. The results clearly reveal that; the increased in compatibility with concentration of EGDMA enhanced transparency for xerogels and hydrogels; this may be explained by the fact that, EGDMA increases compatibility in addition to functioning as a crosslinking agent, yielding enhanced transparency for xerogels. In addition, the improvement of optical homogeneity may be expected from the fact the introduction of EGDMA as a crosslinking agent increases the cross-link density, and hence restricts the mobility of the polymer chain. Figure 4 indicates that translucent xerogel requires only 1% EGDMA for transparency, and the opaque hydrogel requires 1.5% of EGDMA. Light transmission of UV visible spectra confirms these results; an increase in the transmission is expected as the EGDMA increased. A maximum transmission of more than 87% has been achieved through xerogel disks (1 mm thickness).

### Swelling behavior

Table 1 summarizes the swelling properties of five TMSPM30/NVP70 copolymers with different amount of EGDMA within the range (0–2%) in water. All swelling parameters decrease with increasing amount of EGDMA present in the gel formation system. The EWC values are in the range (45.91–52.60). The results clearly reveal that with increasing crosslinker content in the hydrogel, the swelling capacity significantly decreases. The observed results are quite common and may be explained by the fact that the greater number of crosslinks in the hydrogel results in a restrained mobility of the macromolecular chains that does not permit water penetration and brings about a depression in the swelling ratio [20]. Another explanation for the observed finding may be that the increasing number of crosslinks in the hydrogel lowers the molecular weights between the crosslinks [21] and thereby reduces the free volumes between the macromolecular chains, which then become accessible to penetrant water molecules. It was found from Table 1 that the weight loss in the hydrogels (12.22) decreases to its half value (5.77) by the increasing of crosslinker to 2%. The decreasing in ER with increasing EGDMA amount is attributed to the increase of the crosslinking density.

**Table 1:** Swelling parameters of TMSPM30/NVP70 hydrogels with different amount of EGDMA

EGDMA%	EWC%	ER	*ϕ*_2_	*ϕ*_1_	Weight loss%
0.0	52.60	1.22	0.546	0.454	12.22
0.5	50.08	1.19	0.589	0.411	10.55
1.0	48.54	1.17	0.613	0.387	8.11
1.5	47.01	1.15	0.650	0.350	6.82
2.0	45.91	1.13	0.691	0.309	5.77

### Tensile testing

**Fig. 5:**
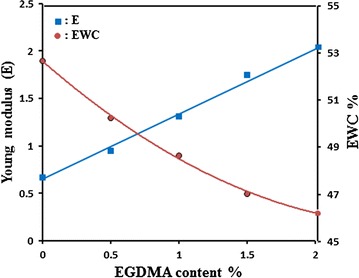
The dependence of EWC % and Young’s modulus for TMSPM30/NVP70 hydrogels system on the concentration of EGDMA

The initial crosslinking concentration also plays an important role in determining mechanical properties of hydrogels. A higher EGDMA concentration generally leads to a stronger and harder gel with lower water content. Results derived from stress–strain measurements as depicted in Table 2 shows that an increase in the concentration of EGDMA results in a concentration increase Young’s and Shear modulus. Young’s moduli are significantly smaller for hydrogels, which exhibit more swelling. Thus, when EGDMA content decreases; the final material is very soft with poor mechanical properties (lower modulus). This indicates that the degree of crosslinking in the network played a major role in the mechanical properties of the hydrogel. As it is well known from Fig. 5, the extent of crosslinking in the network is inversely proportional to the amount of water found on swelling and directly proportional to the Young’s modulus. For an elastic hydrogel, the ratio of ${\text{E}}$ to ${\text{G}}$ should be equal to 3.0 for a small strain. From the set of data in Table 2, the values of ${\text{E}}/{\text{G}}$ do not deviate significantly from the average value of 2.819.

**Table 2:** Tensile properties of TMSPM30/NVP70 hydrogels system containing different concentration of EGDMA

EGDMA%	Young’s moduli (E) (MN/m^2^)	Shear moduli (G) (MN/m^2^)	E/G
0.0	0.669	0.231	2.851
0.5	0.955	0.349	2.736
1.0	1.315	0.476	2.752
1.5	1.751	0.607	2.883
2.0	1.981	0.709	2.828

### Network structures

Determination of share modules (G) allowed the effective crosslinking density ($v_{e}$) to be evaluated; thereby yielding the molar mass between crosslinks (M_c)_. Table 3 contains the effective crosslinking density in the swollen state. Increase crosslinking agent content enhanced hydrophobic bonding and, consequently, the effective crosslink density increased. Table 3 shows also the values of densities, concentration and theoretical crosslinking densities.

**Table 3:** Theoretical network parameters of xerogels containing different concentrations of EGDMA with effective network densities of the swollen gel

EGDMA	P (kg/dm^3^)	Moles (g/mol)	V (xerogel) (dm^3^)	C × 10^−2^ (mol/dm^3^)	*v*_*t*_ (mol/dm^3^)	*v*_*e*_ (mol/dm^3^)
0.0	1.032	–	0.0962	–	–	0.116
0.5	1.051	0.0025	0.0951	2.647	0.052	0.167
1.0	1.048	0.0050	0.0954	5.284	0.105	0.226
1.5	1.072	0.0075	0.0933	8.124	0.162	0.282
2.0	1.066	0.0118	0.0938	12.572	0.251	0.323

In general $v_{e}$ varies with $v_{t}$ according to the following equation [22]:

14 $$v_{e} = \alpha + \beta v_{t}$$

where $\alpha$ is the value of effective cross-linking induced even in the absence of any included chemical crosslinker. It may arise from physical cross-linking, chain transfer, defects in the network and presence of dimethacrylates as an impurity in methacrylates [23].

**Fig. 6:**
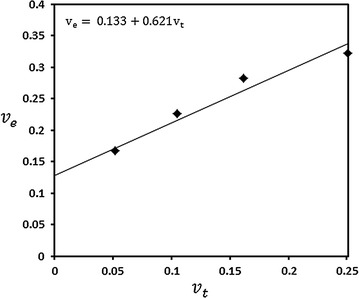
Dependence of measured effective crosslink density ($v_{e}$) on theoretical crosslink density ($v_{t}$) for the gels at different amounts of EGDMA

The parameter $\beta$ is a measure of cross-linking efficiency ($\beta = v_{e} /v_{t}$ when $\alpha = 0$). The linear dependence of $v_{e}$ on $v_{t}$ according to Eq. (13) is indicated in Fig. 6, and by applying a linear least-square fit of the data, the following interrelationship was found:

$$v_{e} = 0.133 + 0.621v_{t}$$

For the present system obtained by thermal polymerization, the values $v_{t}$ being smaller than $v_{e}$. The system BA/VP copolymer which has been prepared by irradiation has shown the same trend, but with a large discrepancy between $v_{e}$ and $v_{t}$ ($v_{e} > v_{t}$) where irradiation contributed to additional chemical crosslinks [24], because poly (acrylates) are known to have a high tendency to crosslink under-irradiation. The average molecular weight between consecutive crosslinks (M_c_) is another structural parameter characterizing the three-dimensional network structure. It is directly related to the crosslink density. The M_c_ values determined for every gel system are given in Table 4. The results obtained show that the average molecular weight between the crosslinks is affected by the concentration of EGDMA and scientifically decreased with increasing the crosslinking concentration.

**Table 4:** Network parameters of TMSPM30/NVP70 hydrogels containing different concentrations of EGDMA

EGDMA %	*ϕ*_2_	Mc × 10^−3^ (g mol^−1^)	χ
0.0	0.546	8.896	0.813
0.5	0.589	6.293	0.860
1.0	0.613	4.637	0.889
1.5	0.650	3.801	0.939
2.0	0.691	3.300	0.987

The polymer–solvent interaction parameter χ at swelling equilibrium represents the specific interaction between water and polymers. Values of χ > 0.50 suggest that the solvent employed is thermodynamically poor. Table 4 reports the values of the polymer–solvent interaction parameter; an increase in EGDMA content led to an increase in χ. This behavior can be explained by the relative hydrophobicity of the EGDMA. All calculated χ values exceeded 0.50, thus an increase in the EGDMA content leads to a reduction in the polymer/water interaction.

### Thermal analysis

**Fig. 7:**
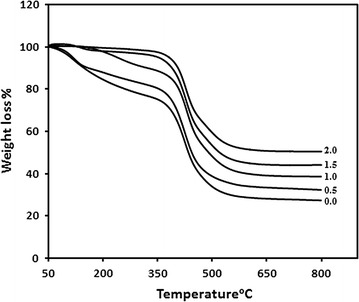
TGA thermogram of xerogels with different amount of EGDMA

In addition to characterization the polymeric sample, the thermal analysis processes provide important information regarding the effect of temperature on sample’s physical properties. Thermal analysis can be used to characterize a polymer before, during, or after crosslinking. The glass transition temperatures (T_g_) were measured for constant composition of TMSPM30/NVP70 xerogels with different concentration of EGDMA (0, 0.5, 1, 1.5 and 2%). The T_g_’s of xerogels were (87.43, 104.48, 110.66, 128.05 and 135.88), respectively. These values are lower than T_g_ of PNVP (172 °C) and higher than T_g_ of PTMSPM (45 °C). As expected, the data revealed that with an increase in EGDMA content, the value of T_g_ increased. The thermal stabilities of the xerogels were determined by (TGA) and are presented in Fig. 7. It is observed that the % weight loss decreased against the temperature by increasing amount of EGDMA in xerogels. The larger amount of a crosslinking agent restricts the segmental mobility of the macromolecular chains, thereby the T_g_ increased and weight loss decreased, this is a common effect of crosslinker on thermal properties of a polymer [25].

### Oxygen permeability

**Fig. 8:**
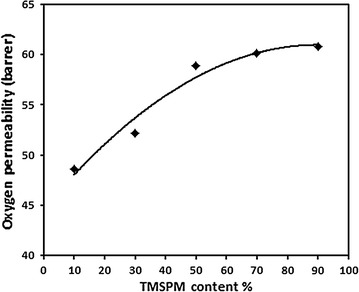
Effect of TMSPM composition on oxygen permeability of TMSPM/NVP hydrogels without EGDMA

The permeability of silicone compounds for such gases as oxygen, making them useful for medical applications in which increased aeration is desired. The enhancement of oxygen permeability in siloxane compounds is associated with high relative proportions of silicon–oxygen and silicon–carbon bonds. These long bonds lead to a free volume element which is greater than other organic compounds [10]. Figure 8 shows the values of oxygen permeability of the prepared hydrogels with different compositions (TMSPM10/NVP90, TMSPM30/NVP70, TMSPM50/NVP50, TMSPM70/NVP30, and TMSPM90/NVP10) without crosslinker, the values are 52.2, 53.9, 58.9, 60.1 and 60.8 barrier, respectively, which are more than oxygen permeability of other non-silicone hydrogels [26, 27] such as poly vinyl pyrrolidone (35.1 barrier), poly hydroxyethyl methacrylate (10.5 barrier) and their copolymer (28 barrier). In addition, the oxygen permeability enhanced as TMSPM composition increased in the feed mixture. For conventional hydrogels, oxygen transport is provided by water contained within the polymer network with an exponential relationship between permeability and EWC. Table 5 shows the relationship between water content and oxygen permeability. An increase of EGDMA decreases the water content of hydrogels and this leads to a reduction in the amount of oxygen permeable. This occurs since oxygen is able to pass through the water rather than through the material itself [28].

**Table 5:** Effect of water content on oxygen permeability of TMSPM30/NVP70 hydrogels containing different concentrations of EGDMA

EGDMA content (%)	Water content (%)	Oxygen permeability (barrier)
0.0	51.2	58.9
0.5	50.0	58.5
1.0	48.6	56.1
1.5	46.4	55.6
2.0	45.7	53.5

## Conclusion

High conversion copolymers were successfully prepared by polymerization mixture of TMSPM–NVP and EGDMA via free radical polymerization using benzoyl peroxide as initiator. Optical clarity enhances with increasing EGDMA. Swelling properties have been determined in deionized water and found that they decrease with addition of EGDMA. Stress–strain measurements yielded the Young’s module, the effective crosslinking density and the copolymer-water interaction parameters. The restriction of chain mobility has been shown by the increase the Young’s modulus of hydrogels and glass transition temperature of xerogels. The oxygen permeability of hydrogels decreases as the water content decrease.

## Abbreviations

TMSPM: 3-(trimethoxysilyl) propyl methacrylate
NVP: *N*-vinyl pyrrolidone
EGDMA: ethylene glycol dimethacrylate
BPO: benzoyl peroxide
FTIR: Fourier transform infrared spectroscopy
TGA: thermogravimetric analysis
DSC: differential scanning calometry
T_g_: glass transition temperature
W_0_: weight of the disc before swelling
W_d_: weight of the disc after drying
W_s_: weight of the swollen disc after 30 days
W_t_: weight of the swollen disc at time t
EWC: equilibrium water content
E: Young’s modulus
G: Shear modulus
τ: the force acting per unit cross-section area
λ: the deformation ratio, deformed length (I)/initial length (I_0_) of hydrogel
ER: extension ratio
d: the diameter of fully hydrated disc
d_0_: the diameter of dried disc
P: permeability
ΔP: pressure through the sample
A: the hydrogel effective area
Q: gas flow
V: volume of Xerogel
C: concentration
ρ: the density of xerogel
ν_e_: effective cross-linking densities of hydrogel
ν_t_: theoretical cross-linking densities of hydrogel
χ: the polymer–solvent interaction parameter at swelling equilibrium
Mc: average molecular weight between consecutive crosslinks
ϕ_1_: the volume fraction of water within the hydrogel at swelling equilibrium
ϕ_2_: the volume fraction of polymer within the hydrogel at swelling equilibrium
